# Diagnostic Accuracy and Renal Safety of Carbon Dioxide Angiography in Lower Limb Angiography: A Systematic Review and Meta-Analysis

**DOI:** 10.3390/diagnostics16203341

**Published:** 2026-10-14

**Authors:** Mosab Fawzy Mossa Alassal, Thamir Alshamari, Arshia Rezaei, Faisal Muhammad Shaikh

**Affiliations:** Department of Vascular Surgery, Lister Hospital, Stevenage SG1 4AB, UK

**Keywords:** carbon dioxide angiography, peripheral arterial disease, iodinated contrast media, contrast-induced nephropathy, diagnostic accuracy, digital subtraction angiography

## Abstract

**Background/Objectives:** Iodinated contrast media (ICM) used in lower limb angiography carry risks of contrast-induced nephropathy (CIN) and allergic reactions, particularly in patients with peripheral arterial disease (PAD) and renal dysfunction. Carbon dioxide (CO_2_) angiography offers a potential non-nephrotoxic alternative. The purpose of this study was to evaluate the diagnostic accuracy and renal safety of CO_2_ angiography compared with ICM in patients undergoing lower limb angiography for PAD. **Methods:** A systematic search of PubMed, Scopus, and Web of Science was performed from inception to 4 September 2025. Studies comparing CO_2_ angiography with ICM in adult PAD patients were included. Risk of bias was assessed with QUADAS-2 for diagnostic accuracy studies and RoB 2 for randomised trials, and the methodological quality of comparative observational studies with the Newcastle–Ottawa Scale. Pooled sensitivity, specificity, adverse events, CIN, contrast volume, serum creatinine, and estimated glomerular filtration rate (eGFR) were analysed using random-effects models. **Results:** Nine studies (527 patients) were included. In three studies (111 patients) with extractable diagnostic accuracy data, CO_2_ angiography showed pooled sensitivity of 0.89 (95% confidence interval [CI]: 0.78–0.95) and specificity of 0.96 (95% CI: 0.85–0.99). Adverse events did not differ significantly between groups (four studies; risk ratio [RR] 0.63, 95% CI: 0.15–2.71), but discomfort during CO_2_ injection was common. CIN risk was lower with CO_2_ (two studies; RR 0.31, 95% CI: 0.14–0.73; *p* = 0.007), and contrast volume was reduced by a mean of 80.71 mL (two studies; *p* < 0.00001). Pooled serum creatinine and eGFR estimates showed extreme heterogeneity (I^2^ = 98–99%), with the two contributing studies reporting effects in opposite directions. **Conclusions:** In a small number of comparative studies, CO_2_ angiography showed pooled sensitivity and specificity compatible with clinically useful diagnostic performance, although these exploratory estimates derive from three studies with unblinded reference standards. The lower risk of CIN with CO_2_, observed in two studies, is hypothesis-generating, and CO_2_ has adverse effects of its own, principally discomfort during injection. These findings should be confirmed in larger prospective trials before CO_2_ can be regarded as an established alternative for patients at risk of CIN or iodine allergy.

## 1. Introduction

### 1.1. Peripheral Arterial Disease and the Need for Angiographic Imaging

Peripheral arterial disease (PAD) is a progressive stenotic and occlusive atherosclerotic condition of the peripheral arteries that most commonly affects the lower extremities. It is a major cause of disability worldwide, producing functional impairment, reduced quality of life, and a substantially increased risk of cardiovascular events and death [1]. The clinical spectrum is wide, extending from asymptomatic arterial narrowing detectable only on physiological testing, through intermittent claudication that limits walking distance, to chronic limb-threatening ischaemia characterised by rest pain, ulceration or gangrene [2,3]. The prevalence of PAD rises steeply with age and is markedly higher among individuals who smoke or who have diabetes mellitus, hypertension or dyslipidaemia.

Accurate anatomical delineation of the arterial tree is central to management once revascularisation is contemplated. Non-invasive modalities including duplex ultrasonography, computed tomography angiography and magnetic resonance angiography each have a role, but catheter-based intra-arterial digital subtraction angiography (DSA) retains a central position, particularly where endovascular treatment is to be undertaken in the same setting. DSA offers high spatial and temporal resolution, permits selective catheterisation of individual vessels, and allows diagnostic imaging and therapeutic intervention to be combined in a single procedure [4].

### 1.2. Iodinated Contrast Media and Contrast-Induced Nephropathy

Iodinated contrast media (ICM) have traditionally been the agents of choice for DSA and provide excellent vascular opacification and spatial resolution [5]. Their use is nonetheless constrained by two principal hazards. The first is hypersensitivity: immediate and delayed reactions to iodinated agents range from mild urticaria to anaphylaxis, and while severe reactions are uncommon, a documented history of hypersensitivity may preclude further exposure [6]. The second, and quantitatively more important in the PAD population, is contrast-induced nephropathy (CIN), an acute deterioration in renal function occurring after intravascular administration of iodinated contrast [7].

The pathophysiology of CIN is incompletely understood but is generally attributed to a combination of direct cytotoxic injury to renal tubular epithelial cells and disturbance of intrarenal haemodynamics, with medullary vasoconstriction producing regional hypoxia in a segment of the nephron that already operates at the margin of its oxygen supply. Osmotic and viscosity effects of the contrast agent contribute, as does the generation of reactive oxygen species. The risk rises with the volume of contrast administered, with intra-arterial rather than intravenous administration, and above all with the degree of pre-existing renal impairment.

This last point is of particular relevance to PAD. Chronic kidney disease (CKD) and PAD share atherosclerotic risk factors and frequently coexist, so that the patients most likely to require repeated angiographic imaging and intervention are also those least able to tolerate the contrast load that such imaging entails. Diabetic patients with chronic limb-threatening ischaemia—a group requiring detailed infrapopliteal imaging and often multiple procedures—represent the clearest expression of this problem. Strategies to reduce iodinated contrast exposure in this population therefore carry direct clinical relevance.

### 1.3. Carbon Dioxide as an Alternative Contrast Agent

Carbon dioxide (CO_2_) angiography was developed as a response to this problem. CO_2_ is a colourless, odourless gas that is neither nephrotoxic nor allergenic. It is approximately twenty times more soluble in blood than oxygen, is rapidly cleared through the pulmonary circulation, and when injected intra-arterially transiently displaces the blood column [8,9]. Because the gas attenuates X-rays far less than blood, the displaced segment appears as a radiolucent column against the surrounding soft tissue, producing a negative contrast image that can be processed by digital subtraction in the same way as a conventional angiogram. The absence of renal excretion means that the agent poses no direct threat to renal function, which makes it attractive in precisely the population in which ICM is most hazardous [10]. CO_2_ is not, however, free of adverse effects: it has a characteristic complication profile of its own, considered in Section 4.4.

CO_2_ has physical characteristics that differ substantially from those of a liquid contrast agent, and these shape both its strengths and its limitations. Its buoyancy causes it to rise within the vessel, so that non-dependent vessel walls are preferentially opacified and patient positioning influences image quality. Its compressibility means that delivery pressure and volume are less easily controlled than with a liquid, and inadequate control may produce either insufficient vessel filling or explosive delivery. Its tendency to fragment into bubbles as it traverses the arterial tree degrades the coherence of the contrast column in smaller distal vessels. These properties explain the recurring observation that CO_2_ performs comparably to ICM in the aortoiliac and femoropopliteal segments while performing less well below the knee, where vessel calibre is smallest and where accurate imaging is often most clinically consequential.

Technique has evolved considerably since CO_2_ angiography was first described. Early series used hand injection from syringes or improvised closed-bag delivery systems, approaches that were inexpensive and widely accessible but offered limited control over delivered volume and pressure and carried a risk of air contamination. Later work employed mechanical injectors and purpose-built delivery circuits, and more recently commercially manufactured automated injectors have been introduced which deliver a pre-set volume of medical-grade CO_2_ at a regulated pressure through a closed system. These developments are relevant to the interpretation of any synthesis spanning several decades, since the index test in an early study and in a contemporary one may differ materially.

### 1.4. Rationale and Objectives

Several comparative studies have examined CO_2_ and ICM in lower limb angiography, reporting variable findings for image quality, diagnostic agreement, safety and renal outcomes [11,12,13]. Two previous systematic reviews have addressed contrast-associated kidney injury with CO_2_ versus ICM in peripheral angiography, and both reported a lower incidence with CO_2_ [14,15]. Neither evaluated diagnostic performance, and to our knowledge no diagnostic test accuracy (DTA) meta-analysis has synthesised the sensitivity and specificity of CO_2_ angiography using ICM angiography as the reference standard.

The present review was undertaken to address that gap and, equally importantly, to characterise how much weight the available evidence can bear. Our objectives were threefold: to pool the available diagnostic accuracy data for CO_2_ angiography against ICM as reference standard; to synthesise the comparative safety and renal outcome data; and to appraise the methodological quality and quantity of the underlying evidence using instruments appropriate to each study design, so that the strength of any conclusion could be judged transparently by the reader.

## 2. Materials and Methods

### 2.1. Protocol and Registration

This systematic review and meta-analysis was conducted and reported in accordance with the Preferred Reporting Items for Systematic Reviews and Meta-Analyses (PRISMA) 2020 statement [16]. A completed PRISMA 2020 checklist is provided as Supplementary Material S1. The protocol was prospectively registered with the International Prospective Register of Systematic Reviews (PROSPERO) under registration number CRD420251138324.

### 2.2. Eligibility Criteria

Eligibility was defined using a PICO framework. The population of interest was adult patients (aged 18 years or over) with lower limb peripheral arterial disease undergoing intra-arterial angiography for diagnostic or therapeutic purposes. The index test or intervention was angiography using CO_2_ as the primary contrast agent. The comparator was angiography using iodinated contrast media. Outcomes of interest were diagnostic accuracy, expressed as sensitivity and specificity or as data from which a two-by-two contingency table could be reconstructed, and safety and tolerability outcomes comprising adverse events, contrast-induced nephropathy, volume of iodinated contrast administered, and post-procedural renal function measured as serum creatinine or estimated glomerular filtration rate.

Randomised controlled trials and prospective or retrospective comparative cohort studies were eligible for inclusion. Studies were excluded if they were non-comparative or single-arm; if they were case reports, narrative reviews, editorials or conference abstracts without an accompanying full text; if they enrolled paediatric populations or used animal models; or if the angiographic procedures studied were unrelated to the lower extremities.

### 2.3. Information Sources and Search Strategy

PubMed, Scopus and Web of Science were searched from database inception to 4 September 2025, the date on which the final search was run; no update search was performed. The search combined controlled vocabulary and free-text terms related to “carbon dioxide angiography”, “iodinated contrast” and “peripheral arterial disease”, combined with Boolean operators and adapted to the syntax of each database (Supplementary Material S2). These three databases provide broad coverage of the biomedical and multidisciplinary literature; Embase, the Cochrane Library, trial registries and grey literature sources were not searched, and this is considered as a limitation in Section 4.7.

### 2.4. Selection Process

**Figure 1:**
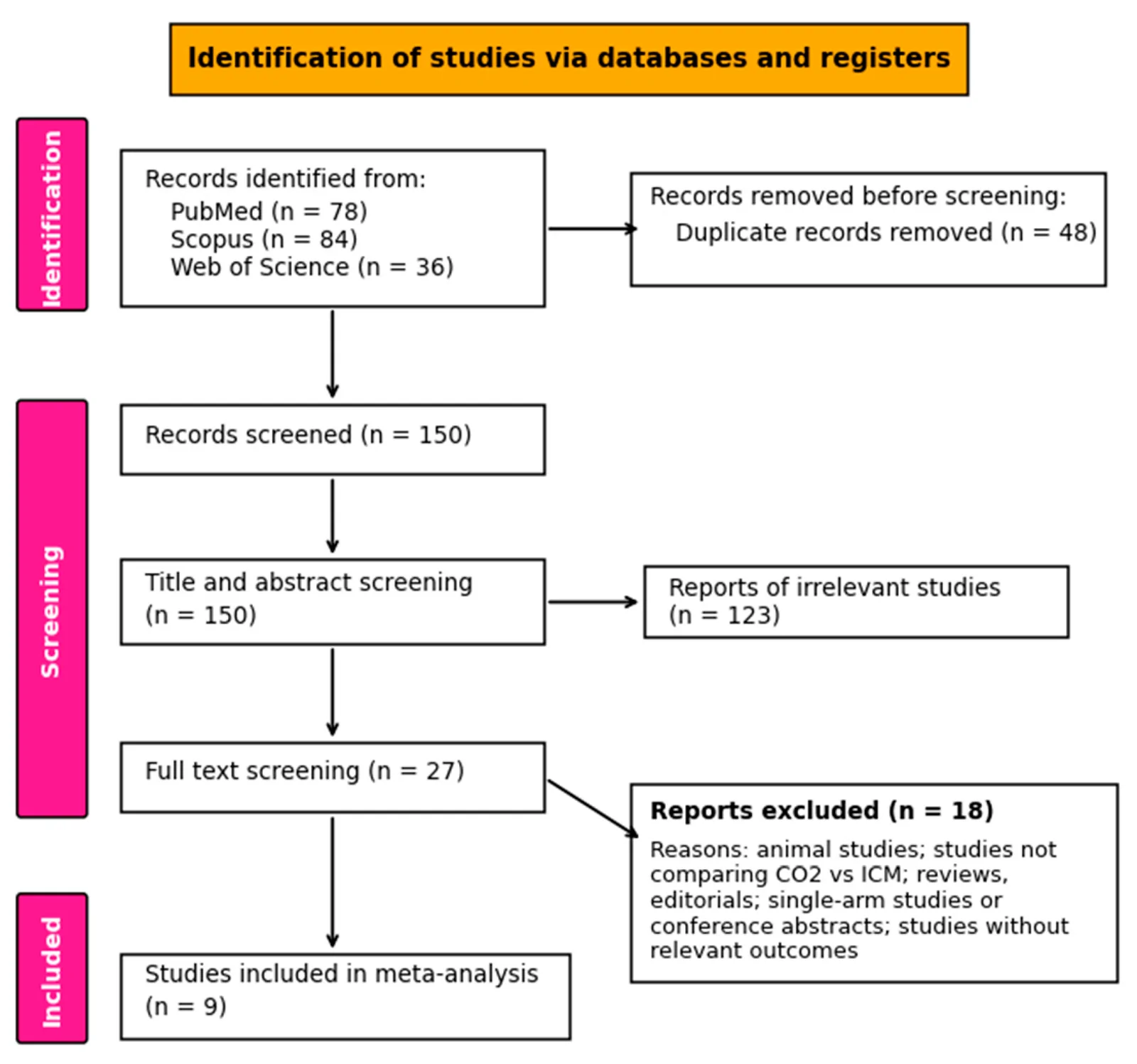
PRISMA 2020 flow diagram of study selection. ICM, iodinated contrast media.

All records retrieved were exported to a reference management application and duplicates were removed. Two reviewers independently screened titles and abstracts against the eligibility criteria. The full texts of all records considered potentially eligible by either reviewer were retrieved and assessed independently in duplicate. Disagreements at either stage were resolved by discussion, and where consensus could not be reached a third reviewer arbitrated. The flow of records through the review is summarised in the PRISMA flow diagram (Figure 1).

### 2.5. Data Extraction

Data were extracted independently in duplicate onto a standardised, piloted extraction form. The following variables were recorded for each included study: first author and year of publication; country and setting; study design; number of participants and, where relevant, number of limbs or arterial segments analysed; participant age and sex distribution; clinical context and indication for angiography; presence of chronic kidney disease or diabetes where reported; the CO_2_ delivery technique used; the comparator agent; the outcomes reported and their definitions; and the principal findings. Diagnostic accuracy data were extracted as two-by-two contingency tables at the patient level, with iodinated contrast angiography as the reference standard. Discrepancies between extractors were resolved by consensus with reference to the original article.

### 2.6. Risk of Bias Assessment

Risk of bias was assessed using the instrument appropriate to each study design and to the outcome under consideration, rather than applying a single tool across all included studies.

For the three studies contributing diagnostic accuracy data, the Quality Assessment of Diagnostic Accuracy Studies 2 (QUADAS-2) tool was applied [17]. Each study was assessed across four domains—patient selection, index test, reference standard, and flow and timing—with risk of bias rated as low, high or unclear in each, and applicability concerns rated separately for the first three domains. Signalling questions were used to support each judgement.

The single randomised controlled trial was assessed using the revised Cochrane risk-of-bias tool for randomised trials (RoB 2) [18], across the domains of bias arising from the randomisation process, bias due to deviations from intended interventions, bias due to missing outcome data, bias in measurement of the outcome, and bias in selection of the reported result, with an overall judgement derived according to the tool’s algorithm.

The remaining comparative observational studies were assessed using the Newcastle–Ottawa Scale (NOS) [19], which evaluates selection of the study groups (maximum four stars), comparability of groups on the basis of design or analysis (maximum two stars), and ascertainment of the outcome of interest (maximum three stars), to a maximum total of nine stars. The NOS is a quality assessment scale rather than a domain-based risk-of-bias instrument and does not specifically assess confounding; the potential for confounding by indication in the non-randomised comparisons was therefore assessed separately on the basis of study design (Section 3.3). All assessments were performed independently by two reviewers and disagreements were resolved by consensus.

### 2.7. Statistical Analysis

Diagnostic accuracy outcomes were pooled using Meta-DiSc version 1.4 (Clinical Biostatistics Unit, Ramón y Cajal Hospital, Madrid, Spain). Sensitivity and specificity were pooled separately using univariate random-effects models. Univariate pooling does not account for the correlation between sensitivity and specificity across studies, which the bivariate random-effects model is designed to address; however, with only three contributing studies this correlation cannot be estimated reliably, and the pooled diagnostic accuracy estimates are therefore presented as exploratory. Individual study estimates and the pooled summary point are displayed in receiver operating characteristic space. In view of the small number of studies, no summary receiver operating characteristic curve is presented and no summary area under the curve is reported.

Safety and renal outcomes were analysed in Review Manager version 5.4 (The Nordic Cochrane Centre, The Cochrane Collaboration, Copenhagen, Denmark) [20]. Dichotomous outcomes were summarised as risk ratios (RR) with 95% confidence intervals using the Mantel–Haenszel method, and continuous outcomes as mean differences (MD) with 95% confidence intervals using the inverse-variance method. A random-effects model was applied throughout, on the basis that clinical and methodological variability between studies was anticipated in advance. Statistical heterogeneity was quantified using the I^2^ statistic, with values of approximately 25%, 50% and 75% taken to indicate low, moderate and high heterogeneity respectively, and was additionally examined using the Cochran Q test. Two-sided *p* values below 0.05 were considered statistically significant.

Formal assessment of publication bias by funnel plot asymmetry or regression-based testing was not undertaken, since such methods are recognised to be uninformative and potentially misleading when fewer than ten studies contribute to an analysis; no outcome in this review met that threshold.

## 3. Results

### 3.1. Study Selection

The electronic search identified 198 records across the three databases: 78 from PubMed, 84 from Scopus and 36 from Web of Science. Following removal of 48 duplicate records, 150 records proceeded to title and abstract screening, of which 123 were excluded as not meeting the eligibility criteria. The full texts of 27 records were assessed in detail. Eighteen were excluded at this stage, for the following reasons: animal studies; studies that did not compare CO_2_ with ICM; reviews and editorials; single-arm studies; conference abstracts without full text; and studies that did not report any outcome of interest. Nine studies met all eligibility criteria and were included in the quantitative synthesis. The selection process is summarised in Figure 1.

### 3.2. Characteristics of Included Studies

The nine included studies were published between 1998 and 2024 and together enrolled 527 participants. Seven were prospective single-centre comparative cohort studies [4,11,12,13,21,22,23], one was an open-label randomised controlled trial [24], and one was a retrospective comparative registry analysis [25]. In the seven prospective studies, both agents were administered to every patient, allowing intra-individual comparison; the randomised trial and the registry compared separate patient groups. The studies were conducted across eight countries, spanning European, Middle Eastern and Asian centres, which provides reasonable geographical breadth although each individual study was single-centre in design.

Sample sizes ranged from 14 to 191 participants. The clinical context varied across studies: some enrolled unselected patients undergoing diagnostic lower limb angiography, whereas others focused specifically on patients with chronic limb-threatening ischaemia, on diabetic patients with critical limb ischaemia and coexisting chronic kidney disease, or on patients with established renal dysfunction in whom avoidance of iodinated contrast was the clinical rationale for CO_2_ use. Mean participant age ranged from 47.6 to 74.8 years, and most cohorts were predominantly male, consistent with the epidemiology of PAD.

CO_2_ delivery technique differed substantially across the included studies and broadly tracked the era of publication. The earliest studies used electronic or hand injection, in one case through a locally improvised closed plastic-bag delivery system [4]. Intermediate-era studies used mechanical injectors with pigtail catheters [21,22]. The four most recent studies used commercially available automated CO_2_ delivery devices, including the Angiodroid system [11,12,23,24]. Full study characteristics are presented in Table 1.

### 3.3. Risk of Bias

Newcastle–Ottawa Scale scores for the eight comparative observational studies ranged from 7 to 9 out of a possible 9, with three studies scoring 9, three scoring 8 and two scoring 7 (Table 2). The item in which stars were most frequently lost was whether follow-up was long enough for outcomes to occur (four studies [4,11,13,21]), reflecting outcome ascertainment restricted to the immediate peri-procedural period. One study also lost a star for assessment of outcome [21], and the retrospective registry received no comparability stars because baseline differences between groups were not adjusted for [25]. No study lost stars in more than one of the three NOS domains; this statement refers to the NOS assessment only, and the QUADAS-2 assessment of the diagnostic accuracy studies, reported below, identified high risk of bias in more than one domain.

Because the NOS does not specifically assess confounding, the potential for confounding by indication was considered separately on the basis of study design. In the seven intra-individual studies, each patient received both agents, so confounding between comparison groups does not arise, although the order of administration and the shared procedural setting may influence comparisons of tolerability. The randomised trial is protected against confounding by randomisation [24]. The retrospective registry [25] compared separate, non-randomised groups, and its authors reported poorer baseline renal function in the CO_2_ group, consistent with preferential use of CO_2_ in patients with renal impairment. As this registry contributes to the analyses of adverse events, contrast-induced nephropathy, contrast volume and post-procedural renal function, these between-group estimates are at substantial risk of confounding by indication.

The randomised controlled trial [24] was assessed using RoB 2. The randomisation process was judged to be at low risk of bias. The domain concerning deviations from intended interventions was rated as raising some concerns, since the open-label design precluded blinding of operators and outcome assessors; this is an inherent constraint of a trial comparing two visibly different contrast agents rather than a deficiency of conduct. Missing outcome data, measurement of the outcome and selection of the reported result were each judged to be at low risk. The overall judgement was therefore “some concerns”.

QUADAS-2 assessment of the three diagnostic accuracy studies is presented in Table 3. In the patient selection domain, all three studies were rated as high or unclear risk, reflecting enrolment at a single centre and restriction to patients already selected for angiography, which introduces the potential for spectrum bias. The index test domain was rated unclear in all three studies because the blinding of index test interpretation was not explicitly described. The reference standard domain was rated as high risk in all three studies: CO_2_ and iodinated contrast angiography were performed within the same procedural session and interpretation of the reference standard was not reported to have been blinded to the index test result, a configuration that tends to inflate apparent agreement between the two modalities. Flow and timing was rated low risk in all three studies, as both tests were applied to all enrolled participants within a single procedure with no partial verification. Applicability concerns were raised for the two studies restricted to critical limb ischaemia and to diabetic patients with CKD, since these populations are narrower than the general PAD population.

### 3.4. Diagnostic Accuracy

**Figure 2:**
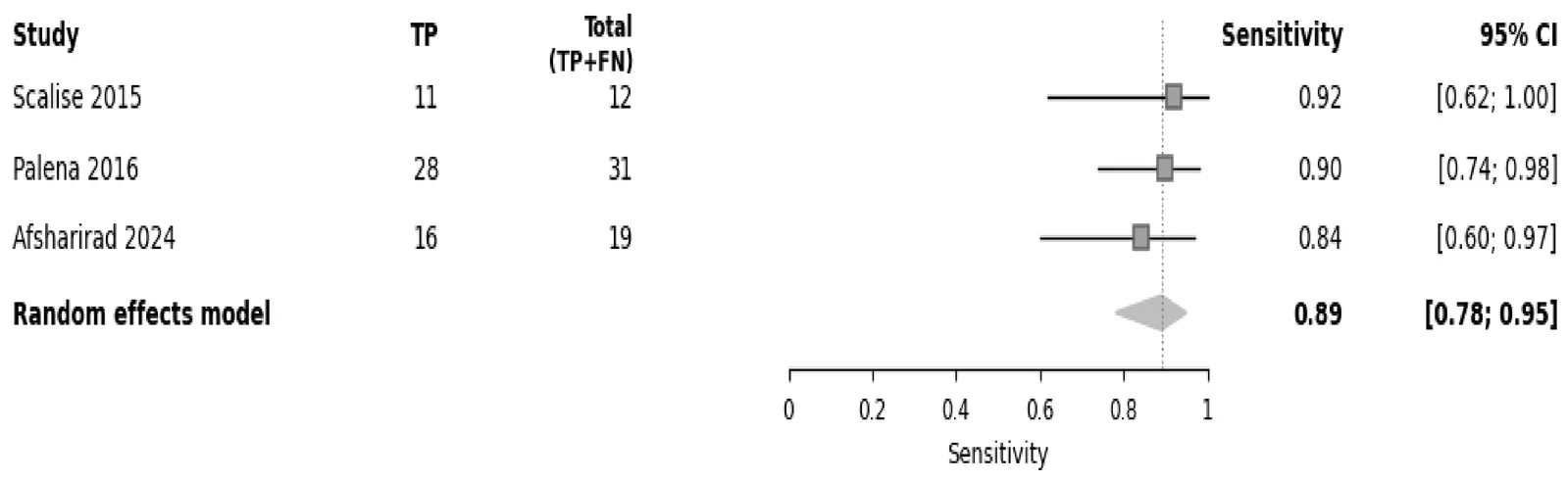
Forest plot of pooled sensitivity of CO_2_ angiography compared with iodinated contrast media [11,12,23]. Grey squares represent individual study estimates and horizontal lines their 95% confidence intervals (CIs); the diamond represents the pooled random-effects estimate, with its width indicating the 95% CI; the dotted vertical line marks the pooled estimate. TP, true positive; FN, false negative.

**Figure 3:**
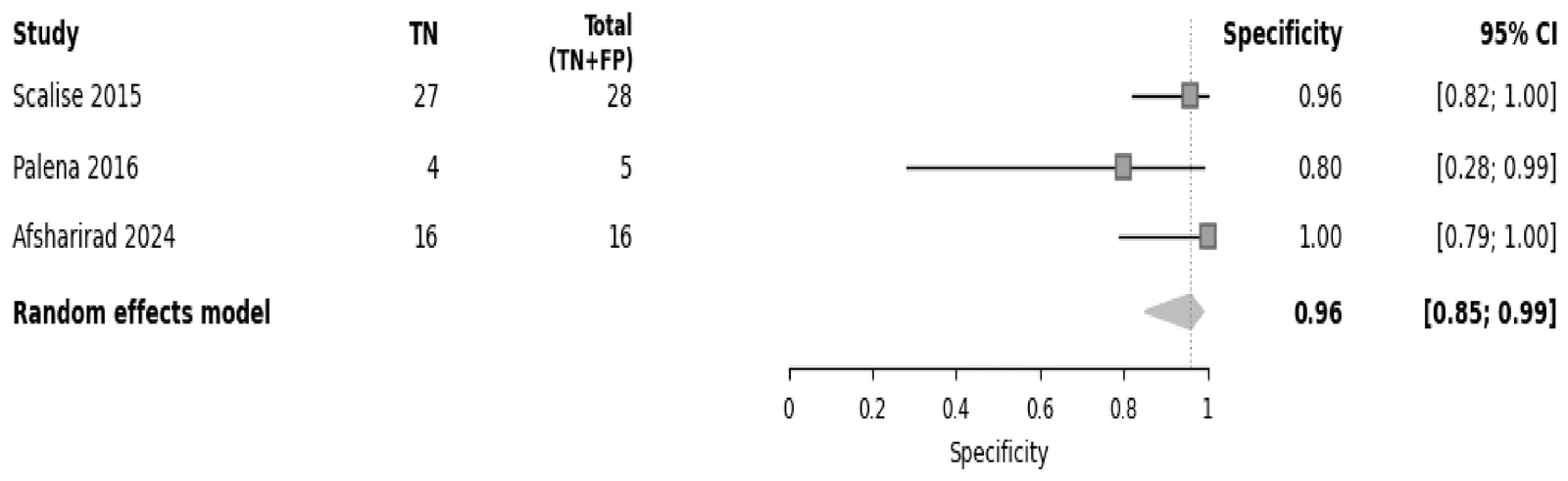
Forest plot of pooled specificity of CO_2_ angiography compared with iodinated contrast media [11,12,23]. Grey squares represent individual study estimates and horizontal lines their 95% confidence intervals (CIs); the diamond represents the pooled random-effects estimate, with its width indicating the 95% CI; the dotted vertical line marks the pooled estimate. TN, true negative; FP, false positive.

**Figure 4:**
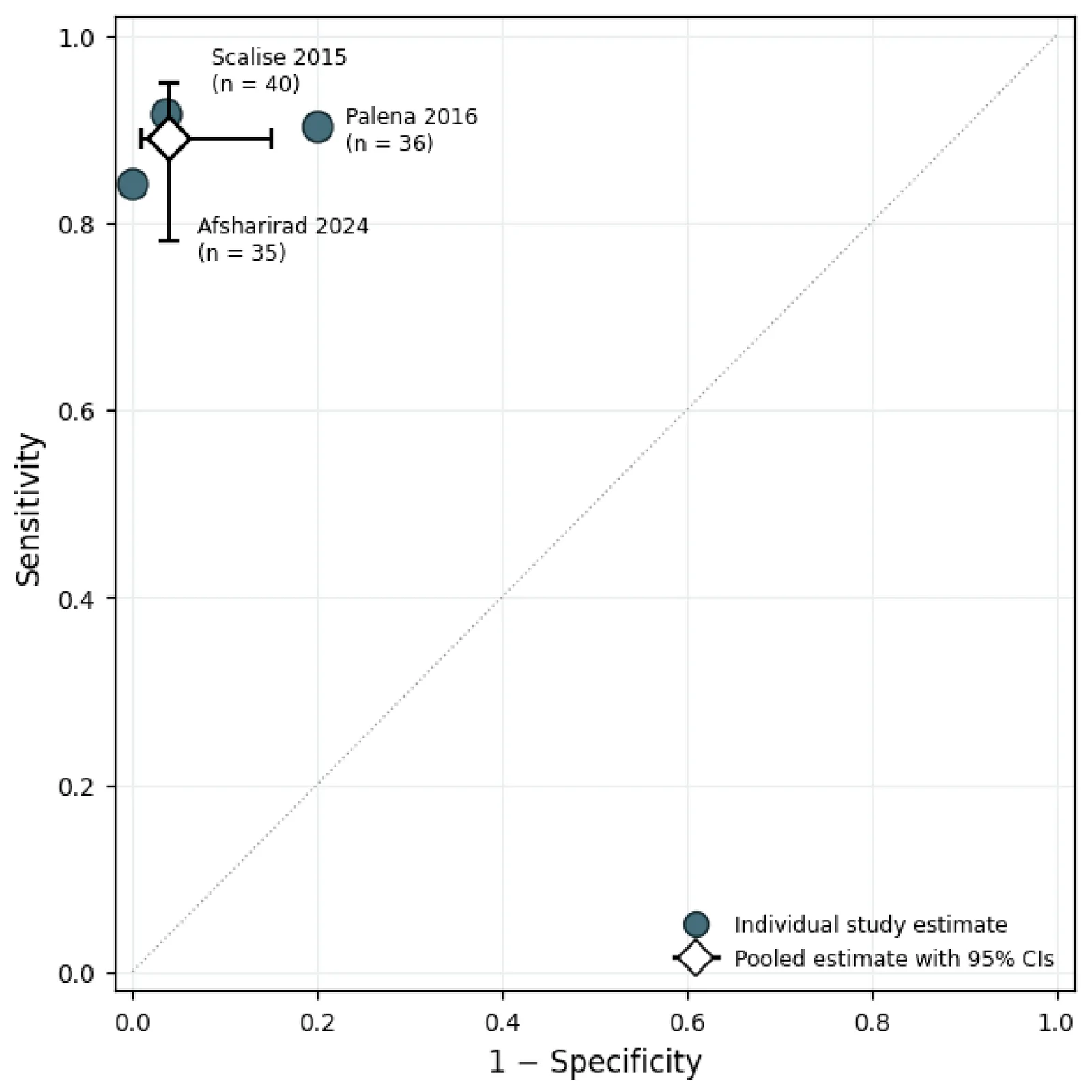
Individual study estimates (circles) and pooled estimate (diamond, with 95% confidence intervals) of the sensitivity and specificity of CO_2_ angiography [11,12,23], plotted in receiver operating characteristic space. No summary receiver operating characteristic curve is fitted, given the small number of studies. The dotted diagonal line represents the line of no discrimination (sensitivity equal to 1 − specificity).

Three studies provided extractable two-by-two data at the patient level [11,12,23], comprising 111 patients, of whom 62 had disease and 49 did not on the reference standard. Individual study sensitivities were 0.92, 0.90 and 0.84, and individual specificities were 0.96, 0.80 and 1.00. Pooled sensitivity was 0.89 (95% CI: 0.78–0.95; Figure 2) and pooled specificity was 0.96 (95% CI: 0.85–0.99; Figure 3). The individual study estimates and the pooled summary point are shown in receiver operating characteristic space in Figure 4; as stated in Section 2.7, no summary receiver operating characteristic curve or area under the curve is reported.

These estimates should be interpreted with considerable caution and regarded as exploratory. They derive from three single-centre studies with a total of 111 patients; the specificity estimate rests on only 49 patients without disease, five of them from a single study [12]; in all three studies, the reference standard was interpreted without documented blinding to the index test and both tests were performed in the same session, a configuration that tends to inflate apparent agreement; and univariate pooling does not account for the correlation between sensitivity and specificity. The pooled estimates are compatible with clinically useful diagnostic performance, particularly in proximal arterial segments, but they do not establish it.

### 3.5. Adverse Events

**Figure 5:**
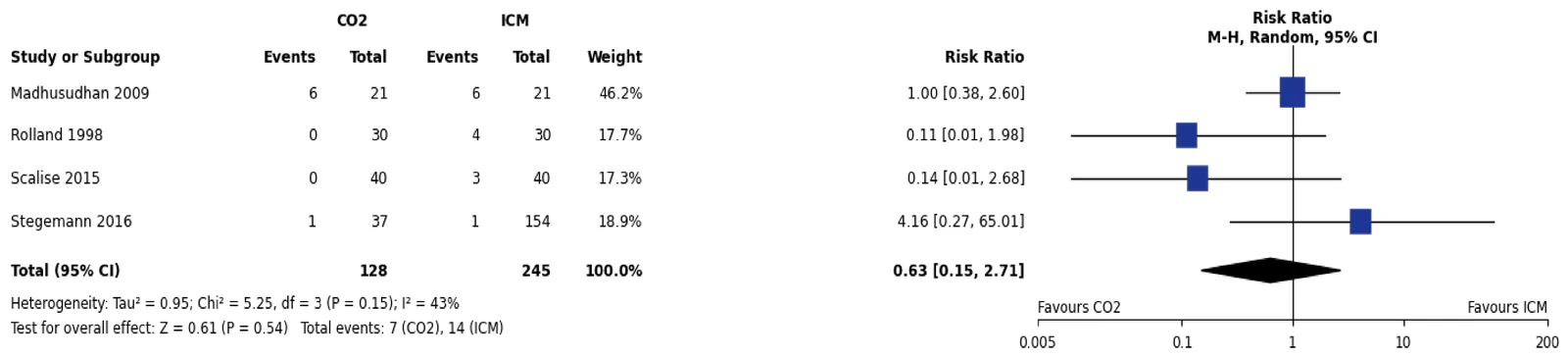
Forest plot of overall adverse events [4,21,23,25]. Coloured squares represent individual study estimates, with size proportional to study weight, and horizontal lines their 95% confidence intervals (CIs); the black diamond represents the pooled random-effects estimate and its 95% CI; the vertical line indicates no difference between groups. ICM, iodinated contrast media; M-H, Mantel–Haenszel.

Four studies reported adverse events in a form that permitted comparison between agents [4,21,23,25], contributing 128 observations in the CO_2_ arm and 245 in the ICM arm; three of these used intra-individual designs [4,21,23]. Seven events were recorded with CO_2_ and fourteen with ICM. The pooled risk ratio was 0.63 (95% CI: 0.15–2.71; *p* = 0.54), with moderate heterogeneity (I^2^ = 43%) (Figure 5). The confidence interval is wide, spanning both a substantial reduction and a substantial increase in risk, and the definition of an adverse event differed between studies; in particular, discomfort during injection was not consistently counted. In one study contributing to this analysis, no adverse events were recorded with CO_2_, although patients rated CO_2_ significantly less comfortable than iodinated contrast [21]. This pooled estimate should therefore not be interpreted as indicating that CO_2_ is better tolerated than iodinated contrast.

CO_2_-specific adverse effects were also reported, although not in a form suitable for pooling. Discomfort or pain during CO_2_ injection was the effect most frequently described. It was reported by 64.3% of patients in one study, in which three of fourteen patients (21.4%) could not tolerate the complete CO_2_ examination [13]; by 48% of patients in another, in which 18% of CO_2_ examinations were discontinued as a result [22]; and in a third study CO_2_ was rated significantly less comfortable than iodinated contrast, with 40% of patients reporting greater discomfort with CO_2_ [21]. In a fourth study, mild pain occurred in 28.6% of patients with each agent, and one patient experienced severe pain during CO_2_ injection [4]. In the two studies using automated injectors that reported on safety, no complications attributable to CO_2_ were reported in one [12], and no allergic reactions or significant decline in renal function were observed with CO_2_ in the other [23]; the retrospective registry reported no severe procedure-related complications [25]. None of the included studies reported vapour lock, symptomatic gas embolism, or mesenteric, coronary or neurological complications.

### 3.6. Contrast-Induced Nephropathy

**Figure 6:**
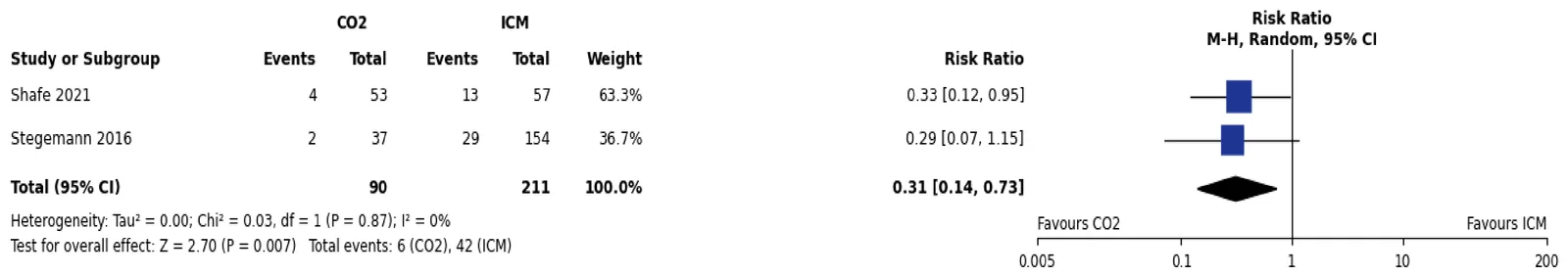
Forest plot of contrast-induced nephropathy (CIN) [24,25]. Coloured squares represent individual study estimates, with size proportional to study weight, and horizontal lines their 95% confidence intervals (CIs); the black diamond represents the pooled random-effects estimate and its 95% CI; the vertical line indicates no difference between groups.

Two studies reported contrast-induced nephropathy as a discrete outcome [24,25], contributing 90 patients in the CO_2_ arm and 211 in the ICM arm. Six CIN events occurred among CO_2_ patients and 42 among ICM patients. The pooled risk ratio was 0.31 (95% CI: 0.14–0.73; *p* = 0.007), with no detectable statistical heterogeneity (I^2^ = 0%) (Figure 6). The two studies were concordant in direction and similar in magnitude despite differing markedly in design, one being a randomised trial and the other a retrospective registry.

This finding should be regarded as hypothesis-generating rather than definitive. It rests on two studies; the definition of CIN and the timing of renal function assessment differed between them, which limits the interpretation of the pooled estimate even in the absence of statistical heterogeneity; and one of the two studies is a retrospective registry at substantial risk of confounding by indication (Section 3.3), although in that study the CO_2_ group had poorer baseline renal function, a bias that would be expected to work against, rather than in favour of, CO_2_.

### 3.7. Iodinated Contrast Volume

**Figure 7:**
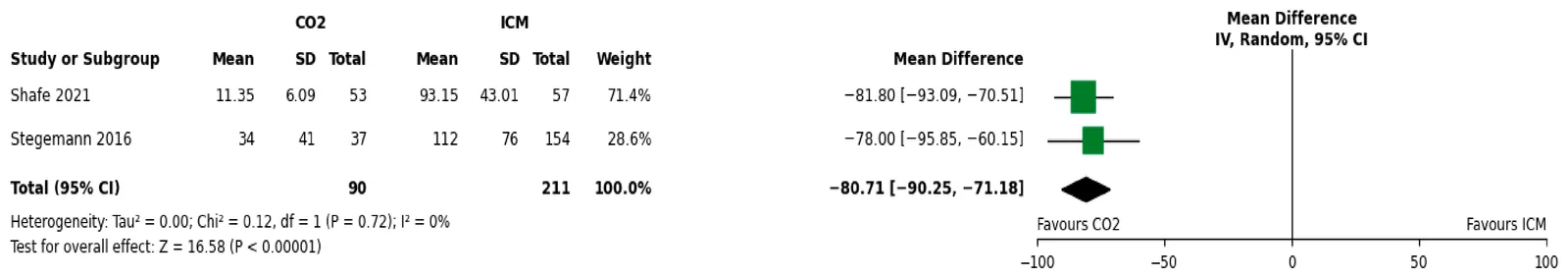
Forest plot of contrast media volume (mL) [24,25]. Coloured squares represent individual study estimates, with size proportional to study weight, and horizontal lines their 95% confidence intervals (CIs); the black diamond represents the pooled random-effects estimate and its 95% CI; the vertical line indicates no difference between groups. IV, inverse variance; SD, standard deviation.

The same two studies reported the volume of iodinated contrast administered [24,25]. Volume was substantially lower in the CO_2_ arm, with a pooled mean difference of −80.71 mL (95% CI: −90.25 to −71.18; *p* < 0.00001) and no detectable heterogeneity (I^2^ = 0%) (Figure 7). This finding confirms that the intended intervention was delivered as planned; it is in part a function of protocol design, since a strategy specifying CO_2_ as the primary agent necessarily reduces iodinated contrast exposure.

### 3.8. Post-Procedural Renal Function

Two studies reported serum creatinine and estimated glomerular filtration rate (eGFR) on the second post-procedural day [24,25]. The randomised trial reported lower creatinine (mean difference [MD] −0.17 mg/dL, 95% CI −0.29 to −0.04) and higher eGFR (MD 9.92 mL/min/1.73 m^2^, 95% CI 4.17 to 15.67) with CO_2_ than with ICM [24]. The retrospective registry reported higher creatinine (MD 1.00 mg/dL, 95% CI 0.69 to 1.31) and lower eGFR (MD −32.00 mL/min/1.73 m^2^, 95% CI −37.53 to −26.47) in the CO_2_ group [25], in which renal function was poorer at baseline.

**Figure 8:**
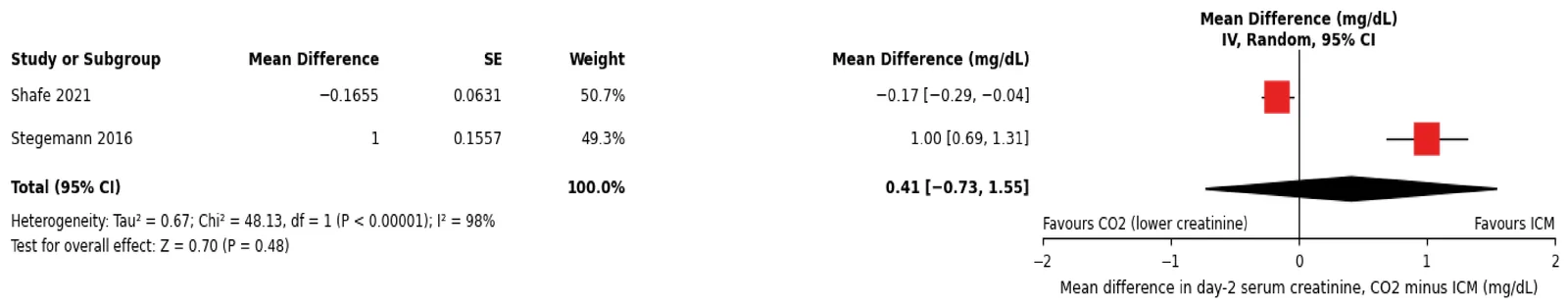
Forest plot of postprocedural serum creatinine at day 2 [24,25] (mean difference, CO_2_ minus ICM, in mg/dL). The pooled estimate is shown for completeness only; because the two studies report effects in opposite directions with extreme heterogeneity (I^2^ = 98%), it should not be used for inference. Coloured squares represent individual study estimates, with size proportional to study weight, and horizontal lines their 95% confidence intervals (CIs); the black diamond represents the pooled random-effects estimate and its 95% CI; the vertical line indicates no difference between groups.

**Figure 9:**
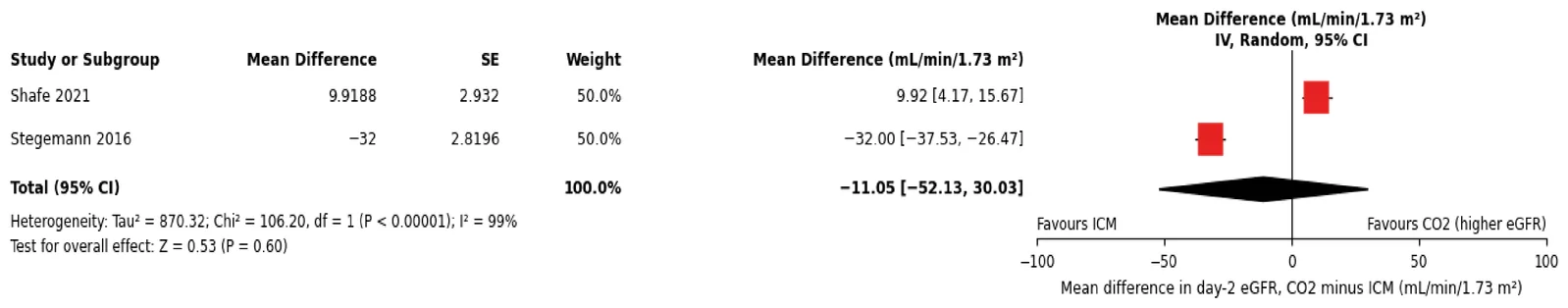
Forest plot of postprocedural estimated glomerular filtration rate (eGFR) at day 2 [24,25] (mean difference, CO_2_ minus ICM, in mL/min/1.73 m^2^); positive values favour CO_2_. The pooled estimate is shown for completeness only; because the two studies report effects in opposite directions with extreme heterogeneity (I^2^ = 99%), it should not be used for inference. Coloured squares represent individual study estimates, with size proportional to study weight, and horizontal lines their 95% confidence intervals (CIs); the black diamond represents the pooled random-effects estimate and its 95% CI; the vertical line indicates no difference between groups.

For completeness, pooled estimates are shown in Figure 8 (serum creatinine: MD 0.41 mg/dL, 95% CI −0.73 to 1.55; *p* = 0.48; I^2^ = 98%) and Figure 9 (eGFR: MD −11.05 mL/min/1.73 m^2^, 95% CI −52.13 to 30.03; *p* = 0.60; I^2^ = 99%). Because the two studies reported effects in opposite directions with extreme heterogeneity, these pooled estimates do not represent a meaningful average effect and should not be used for inference. Post-procedural values measured at day 2 also reflect baseline renal function and may not capture delayed renal injury.

## 4. Discussion

### 4.1. Principal Findings

This systematic review synthesised nine comparative studies enrolling 527 patients and examined both the diagnostic performance and the safety of CO_2_ angiography relative to iodinated contrast angiography in the lower limb. Four findings emerge. First, in the three studies providing extractable diagnostic data, pooled sensitivity was 0.89 and specificity 0.96 against ICM as reference standard; given the small number of studies, the unblinded reference standard and the use of univariate pooling, these estimates are exploratory. Second, CO_2_ was associated with a lower incidence of contrast-induced nephropathy in two studies, a hypothesis-generating finding, and with a large reduction in iodinated contrast volume. Third, overall adverse event rates did not differ significantly between agents, but this analysis was imprecise and did not consistently capture discomfort during injection. Fourth, CO_2_ has characteristic adverse effects of its own: discomfort during injection was common, and in some studies severe enough to prevent completion of the examination.

These findings should be read in the context of the evidence base from which they derive. The diagnostic accuracy synthesis rests on three studies, and the CIN and contrast volume analyses on two each. No outcome in this review was informed by more than four studies. The conclusions that follow are therefore appropriately regarded as provisional.

### 4.2. Mechanistic Basis for the Renal Findings

The renal safety advantage observed for CO_2_ has a coherent mechanistic explanation. Iodinated contrast agents are excreted almost entirely by glomerular filtration and come into direct contact with tubular epithelium at high concentration, where they exert cytotoxic effects and contribute to medullary hypoxia through vasoconstriction and increased viscosity of tubular fluid. CO_2_, by contrast, is not filtered by the kidney at all; it dissolves in blood, is transported to the pulmonary circulation and is eliminated in expired gas, typically within a single circulation time. The renal tubule is therefore never exposed to the agent. On this basis a reduction in CIN with CO_2_ is biologically plausible, and the direction of the pooled estimate is consistent with the two previously published syntheses addressing this question [14,15], although in the present review the clinical evidence for it is limited to two studies.

An important qualification applies. In most reported protocols, CO_2_ is used as the primary rather than the exclusive contrast agent, with small volumes of iodinated contrast retained for selected runs where CO_2_ imaging is inadequate—most often below the knee. What such protocols achieve is a substantial reduction in iodinated contrast dose rather than its complete elimination, and the renal benefit observed should be understood in those terms.

### 4.3. Diagnostic Performance by Anatomical Segment

The pooled sensitivity of 0.89 conceals a pattern that is consistent across the narrative findings of the included studies. CO_2_ angiography performs comparably to iodinated contrast in the aortoiliac and femoropopliteal segments, and less well in the infrapopliteal and pedal vessels. Several studies commented explicitly on degraded image quality below the knee [4,13,21], and at least one concluded that CO_2_ could not replace iodinated contrast for routine lower extremity imaging on the basis of distal performance [22].

This gradient is explicable from the physical behaviour of the gas. Vessel calibre decreases distally, and the coherence of the CO_2_ column degrades as it fragments into smaller bubbles during transit. Buoyancy effects become proportionally more significant in smaller vessels, and the shorter transit time available for image acquisition compounds the difficulty. The clinical significance of this limitation is considerable, because infrapopliteal disease is precisely what determines revascularisation strategy in chronic limb-threatening ischaemia, and is most prevalent in diabetic patients with CKD—the group for whom CO_2_ is otherwise most attractive.

The more recent studies using automated delivery systems reported better distal opacification than the earlier manual series [11,12,23]. Whether this reflects genuine technological improvement, differences in patient selection, or greater operator familiarity cannot be determined from the available data, since delivery technique is confounded with publication era across this literature.

### 4.4. Technical Considerations and CO_2_-Specific Complications

CO_2_ angiography requires attention to technique in ways that liquid contrast angiography does not. Delivered volume and injection pressure must be controlled to obtain adequate opacification without excessive gas administration, and an interval between injections is conventionally observed to permit clearance and avoid accumulation. Closed delivery systems are important to prevent contamination with room air, since nitrogen is far less soluble than CO_2_ and is not cleared with the same rapidity.

Just as contrast-induced nephropathy is characteristic of iodinated contrast, CO_2_ has a characteristic complication profile of its own [8,9,26]. Minor and usually transient effects include pain or discomfort at and distal to the injection site, abdominal pain, nausea and vomiting, paraesthesiae and tenesmus; the abdominal and rectal symptoms are attributed to gas refluxing into, and transiently trapped within, the mesenteric circulation. Major complications are uncommon but potentially serious. Vapour lock occurs when a volume of gas accumulates in a non-dependent vessel segment or cardiac chamber and obstructs forward flow, most consequentially in the right ventricular outflow tract or pulmonary artery, where it can cause hypotension and cardiovascular collapse. Gas reaching the cerebral circulation can cause neurological injury, which underlies the contraindication to CO_2_ injection above the diaphragm and in patients with right-to-left intracardiac or pulmonary shunts, and gas trapped within the coronary or mesenteric circulations can cause myocardial or bowel ischaemia. Contamination of the delivery system with room air increases these risks, because nitrogen is far less soluble than CO_2_ and is cleared much more slowly.

Among the included studies, the CO_2_-specific effects reported were predominantly discomfort and pain during injection (Section 3.5). These were common in the earlier series, most of which used electronic or mechanical injection: discomfort affected around half or more of patients in two studies, led to discontinuation of 18% of CO_2_ examinations in one, and was significantly greater with CO_2_ than with iodinated contrast in another [13,21,22]. Studies using automated delivery systems reported few or no CO_2_-related complications [12,23], consistent with the view that much of the morbidity of CO_2_ angiography is related to technique, although delivery system is confounded with publication era across this literature. No included study reported vapour lock, symptomatic gas embolism, or mesenteric, coronary or neurological complications. The included studies were small, however, and were not designed or powered to detect rare events; the absence of reported major complications should therefore not be taken as evidence that the risk is negligible.

The safety comparison between CO_2_ and iodinated contrast is therefore not one-sided. CO_2_ avoids the nephrotoxicity and hypersensitivity risks of iodinated contrast but introduces risks of its own: chiefly procedural discomfort, which in some series was sufficient to prevent completion of the examination, and rare but potentially serious gas-related complications that the available comparative studies could not quantify. These risks are mitigated by appropriate patient selection, restriction of injection to below the diaphragm, closed delivery systems that exclude room air, controlled injection volumes with adequate intervals between injections, patient positioning that limits gas trapping in non-dependent vessels, and adequate analgesia.

### 4.5. Comparison with Previous Work

Two prior meta-analyses have addressed contrast-associated kidney injury following peripheral angiography with CO_2_ compared with iodinated agents, and both reported findings in the same direction as ours [14,15]. We do not claim novelty for the renal safety result, which is best understood as corroboration from a partially overlapping study set. The contribution that distinguishes the present review is the diagnostic accuracy synthesis, which to our knowledge has not previously been attempted, together with the explicit appraisal of methodological quality using instruments matched to study design.

The value of the latter deserves emphasis. Applying QUADAS-2 to the diagnostic accuracy studies revealed a consistent and material limitation that a generic quality instrument would not have surfaced: in all three studies, the reference standard was interpreted without documented blinding to the index test result, and both tests were performed in a single session. In paired imaging comparisons, this configuration tends to inflate measured agreement, and it provides a specific reason for caution in interpreting the pooled accuracy estimates beyond the simple limitation of small study numbers.

### 4.6. Clinical Implications

Taken together, the available evidence supports a defined rather than a general role for CO_2_ angiography in the lower limb. In patients for whom iodinated contrast exposure is itself the principal hazard—those with advanced chronic kidney disease, those with documented iodinated contrast hypersensitivity, and those requiring repeated procedures with cumulative contrast exposure—CO_2_ offers a means of obtaining diagnostic imaging while substantially reducing that exposure, and the limited available data suggest a corresponding reduction in contrast-induced nephropathy.

The evidence does not support the wider claim that CO_2_ is diagnostically equivalent to iodinated contrast across the lower limb arterial tree, and it would be premature to treat it as a general substitute. A pragmatic approach consistent with the data would use CO_2_ as the primary agent for aortoiliac and femoropopliteal imaging in at-risk patients, with small-volume iodinated contrast reserved for infrapopliteal runs where CO_2_ imaging proves inadequate. Such a strategy retains most of the potential renal benefit while mitigating the principal diagnostic limitation. Patient selection should also take account of the contraindications to CO_2_ and of procedural discomfort, which may require analgesia.

### 4.7. Strengths and Limitations

The strengths of this review include a protocol registered prospectively with PROSPERO, duplicate independent screening and data extraction, the application of risk-of-bias instruments matched to study design rather than a single generic tool, and the explicit reporting of the number of contributing studies and participants alongside every pooled estimate.

Several limitations must nonetheless be acknowledged, and the first is the most important. The evidence base is small and unevenly distributed across outcomes: only three studies and 111 patients contributed to the diagnostic accuracy analysis, and only two studies each to the contrast-induced nephropathy, contrast volume and renal function analyses. Conclusions for these outcomes should be regarded as preliminary rather than definitive.

Second, only one of the nine included studies was randomised. Risk of bias in the non-randomised studies was appraised with the Newcastle–Ottawa Scale, which is a quality assessment scale rather than a formal risk-of-bias instrument and does not specifically address confounding; a domain-based tool such as ROBINS-I was not applied. Seven of the eight non-randomised studies used intra-individual designs, in which confounding between comparison groups does not arise, but the one retrospective registry, which contributes to every between-group safety analysis, compared non-randomised groups with poorer baseline renal function in the CO_2_ group, and its estimates are at substantial risk of confounding by indication. This bias would be expected to work against the observed benefit of CO_2_ for CIN, but in the opposite direction for absolute post-procedural renal function values. In addition, adverse events in the three intra-individual studies contributing to the adverse event analysis were analysed as if arising from independent groups, which does not account for the pairing of observations within patients.

Third, with only three studies, the correlation between sensitivity and specificity could not be estimated reliably; univariate pooling does not account for it or for any threshold effect and will tend to understate uncertainty. Fourth, blinded interpretation of the reference standard was absent or unreported in all three diagnostic accuracy studies. Fifth, the included studies span twenty-six years and a correspondingly wide range of CO_2_ delivery technology, so pooled estimates may not describe the performance of any single contemporary technique. Sixth, the definition of contrast-induced nephropathy and the timing of renal function assessment varied between studies, and assessment at day two may fail to capture delayed injury. Seventh, the definition of adverse events varied between studies, discomfort during CO_2_ injection was not consistently counted, and the included studies were not designed to detect rare CO_2_-specific complications such as vapour lock or gas embolism. Eighth, publication bias could not be formally assessed given the small number of contributing studies. Ninth, only PubMed, Scopus and Web of Science were searched; Embase, the Cochrane Library, trial registries and grey literature were not, and relevant studies, particularly European observational studies and unpublished trials, may have been missed. Tenth, the search was completed on 4 September 2025 and was not updated, and studies published since then are not represented.

### 4.8. Directions for Future Research

The clearest need is for adequately powered randomised evidence comparing CO_2_ with iodinated contrast in patients with PAD and coexisting chronic kidney disease, with renal function as a pre-specified primary outcome assessed at intervals sufficient to capture delayed injury rather than at day two alone. For diagnostic accuracy, what is required is a prospective study with blinded and independent interpretation of index and reference standard images by separate readers, pre-specified analysis stratified by arterial segment above and below the knee, and the use of a contemporary automated delivery system, so that the resulting estimates describe a technique in current clinical use. Standardisation of CIN definitions across studies, and consistent reporting of CO_2_ delivery parameters including system, volume and injection pressure, would materially improve the interpretability of future syntheses in this field. Comparative studies should also record CO_2_-specific complications prospectively and systematically, using standardised definitions, so that the safety of the two agents can be compared on equal terms.

## 5. Conclusions

In the comparative studies available to date, CO_2_ angiography showed pooled sensitivity and specificity, relative to iodinated contrast angiography in the lower limb, that are compatible with clinically useful diagnostic performance, although these estimates are exploratory: they derive from three small studies with unblinded reference standards. Diagnostic performance appears best in the aortoiliac and femoropopliteal segments and less reliable below the knee, a limitation of direct clinical relevance in chronic limb-threatening ischaemia. CO_2_ was associated with a lower incidence of contrast-induced nephropathy, a hypothesis-generating finding based on two studies, and with a substantial reduction in iodinated contrast volume.

CO_2_ is not without risk: discomfort during injection was common in the included studies and occasionally prevented completion of the examination, and rare but serious gas-related complications, although not reported in the included studies, are recognised. On present evidence, CO_2_ angiography is best positioned as a contrast-sparing strategy for carefully selected patients in whom iodinated contrast exposure poses a specific hazard, rather than as a general alternative to iodinated contrast angiography. Larger prospective randomised trials, and diagnostic accuracy studies employing blinded reference standard interpretation, contemporary delivery systems and systematic recording of CO_2_-specific complications, are required before firmer conclusions can be drawn.

## Figures and Tables

**Table 1:** Summary of the studies included.

Study (Year)	Design (Country)	Participants	Age	M/F	CO_2_ Technique	Principal Findings
Rolland 1998 [21]	Prospective (France)	30 limbs/30 pts	67	20/10	Electronic injection	Equal to iodinated in pelvis, poorer below knee
Díaz 2000 [22]	Prospective, double-blind review (Spain)	50 pts	65	42/8	Mechanical injector	Could not replace iodinated contrast
Ho 2003 [13]	Prospective (Taiwan)	14 pts	74.6	9/5	Mechanical injector	Acceptable in thigh, sub-optimal below knee
Madhusudhan 2009 [4]	Prospective (India)	21 pts/27 limbs	47.6	21/0	Hand, improvised system	86% adequate opacification of major vessels
Scalise 2015 [23]	Prospective (Italy)	40 CLI pts	71.7	24/16	Angiodroid injector	Accurate and kidney-sparing in CLI
Palena 2016 [12]	Prospective (Italy)	36 diabetic CKD/CLI	74.8	27/9	Automated (ACDA)	Safe and effective for infrapopliteal work
Stegemann 2016 [25]	Retrospective (Germany)	191 pts	70.6	141/50	Manual/assist pump	Reduced iodinated load and CIN
Shafe 2021 [24]	Open-label RCT (Egypt)	110 pts	62.9	86/24	Automated (Angiodroid)	Reduced contrast dose and CIN
Afsharirad 2024 [11]	Prospective (Iran)	35 pts	56.9	19/16	Automatic device	High diagnostic accuracy of modern CO_2_

ACDA = automated carbon dioxide angiography; CLI = critical limb ischemia; CIN = contrast-induced nephropathy; CKD = chronic kidney disease; pts = patients; RCT = randomized controlled trial.

**Table 2:** Newcastle–Ottawa Scale assessment of comparative observational studies.

Study	Selection (4)	Comparability (2)	Outcome (3)	Total
Afsharirad 2024 [11]	4	2	2	8/9
Díaz 2000 [22]	4	2	3	9/9
Ho 2003 [13]	4	2	2	8/9
Madhusudhan 2009 [4]	4	2	2	8/9
Palena 2016 [12]	4	2	3	9/9
Rolland 1998 [21]	4	2	1	7/9
Scalise 2015 [23]	4	2	3	9/9
Stegemann 2016 [25]	4	0	3	7/9

Shafe 2021 [24], a randomized controlled trial, was assessed with the Cochrane RoB 2 tool: randomization process—low risk; deviations from intended interventions—some concerns (open-label design); missing outcome data—low risk; measurement of the outcome—low risk; selection of the reported result—low risk. Overall judgement: some concerns.

**Table 3:** QUADAS-2 assessment of diagnostic accuracy studies.

Study	Patient Selection	Index Test	Reference Standard	Flow and Timing	Applicability
Scalise 2015 [23]	High	Unclear	High	Low	Concerns: CLI only
Palena 2016 [12]	High	Unclear	High	Low	Concerns: diabetic CKD/CLI only
Afsharirad 2024 [11]	Unclear	Unclear	High	Low	Moderate concerns

The reference standard domain was rated high risk in all three studies because CO_2_ and ICM angiography were performed within the same session and blinded interpretation of the reference standard was not described.

## Data Availability

All data analysed during this study are derived from the published articles cited in the reference list and are presented within this article and its Supplementary Materials. No new data were created.

## Supplementary Materials

The following supporting information can be downloaded at: https://www.mdpi.com/article/10.3390/diagnostics16203341/s1, Supplementary Material S1, PRISMA 2020 Checklist; Supplementary Material S2, Full Electronic Search Strategies.
