# Supplementary material for: Diagnostic Accuracy and Renal Safety of Carbon Dioxide Angiography in Lower Limb Angiography: A Systematic Review and Meta-Analysis
Source: Diagnostics (Basel). 2026 Oct 14;16(20):3341. doi: 10.3390/diagnostics16203341 (PMC13702889; doi:10.3390/diagnostics16203341)
Supplement: Supplementary file 1 [file diagnostics-16-03341-s001.zip › diagnostics-4598927 Supplementary_S1_PRISMA_2020_Checklist.pdf]

Supplementary Material S1. PRISMA 2020 Checklist

Manuscript: Diagnostic Accuracy and Renal Safety of Carbon Dioxide Angiography in Lower Limb Angiography: A Systematic Review and Meta-Analysis

Section and topic references refer to the numbered sections of the revised manuscript. Items not performed are stated as such, with the reason given, in accordance with PRISMA 2020 guidance that reviewers should report what was done rather than what was intended.

**Item**	**Checklist item**	**Location where item is reported**
TITLE		
1. Title	Identify the report as a systematic review.	Title page — title states "A Systematic Review and Meta-Analysis".
ABSTRACT		
2. Abstract	Report an abstract addressing each item in the PRISMA 2020 for Abstracts checklist.	Abstract (structured: Background/Objectives, Methods, Results, Conclusions).
INTRODUCTION		
3. Rationale	Describe the rationale for the review in the context of existing knowledge.	Section 1.1–1.4.
4. Objectives	Provide an explicit statement of the objective(s) or question(s) the review addresses.	Section 1.4, final paragraph.
METHODS		
5. Eligibility criteria	Specify the inclusion and exclusion criteria and how studies were grouped for the syntheses.	Section 2.2.
6. Information sources	Specify all databases, registers, websites, organisations, reference lists and other sources searched, and the date when each was last searched.	Section 2.3 (PubMed, Scopus, Web of Science; inception to July 2024; reference lists hand-searched).
7. Search strategy	Present the full search strategies for all databases, registers and websites, including any filters and limits used.	Section 2.3 and Supplementary Material S2.
8. Selection process	Specify the methods used to decide whether a study met the inclusion criteria, including how many reviewers screened each record, whether they worked independently, and details of automation tools used.	Section 2.4 (two independent reviewers; third-reviewer arbitration; no automation tools used).
9. Data collection process	Specify the methods used to collect data from reports, including how many reviewers collected data, whether they worked independently, and details of automation tools used.	Section 2.5 (duplicate independent extraction on a piloted form; no automation tools used).
10a. Data items (outcomes)	List and define all outcomes for which data were sought.	Section 2.2 (Outcomes) and Section 2.5.
10b. Data items (other variables)	List and define all other variables for which data were sought.	Section 2.5.
11. Study risk of bias assessment	Specify the methods used to assess risk of bias, including details of the tool(s) used and how many reviewers assessed each study.	Section 2.6 (QUADAS-2, RoB 2, Newcastle-Ottawa Scale; duplicate independent assessment).
12. Effect measures	Specify for each outcome the effect measure(s) used in the synthesis or presentation of results.	Section 2.7 (risk ratios; mean differences; pooled sensitivity and specificity).
13a. Synthesis methods (eligibility for synthesis)	Describe the processes used to decide which studies were eligible for each synthesis.	Sections 2.2 and 2.7.
13b. Synthesis methods (data preparation)	Describe any methods required to prepare the data for presentation or synthesis.	Section 2.5 (reconstruction of 2×2 tables with independent checking) and Section 2.7 (continuity correction).
13c. Synthesis methods	Describe any methods used to tabulate or visually display results of individual studies and syntheses.	Tables 1–3; Figures 1–9.

(tabulation/visual display)		
13d. Synthesis methods (statistical synthesis)	Describe any methods used to synthesise results and provide a rationale for the choice(s); identify the model(s) and method(s) used.	Section 2.7 (random-effects throughout; Mantel-Haenszel and inverse-variance; univariate pooling for DTA with rationale and stated limitation).
13e. Synthesis methods (heterogeneity)	Describe any methods used to explore possible causes of heterogeneity among study results.	Section 2.7 (I^2, Cochran Q); Sections 4.3 and 4.7 (narrative exploration: delivery technique, baseline renal function, timing of assessment).
13f. Synthesis methods (sensitivity analyses)	Describe any sensitivity analyses conducted to assess robustness of the synthesised results.	Not performed. The small number of studies per outcome (two to four) precluded meaningful sensitivity analysis; stated in Section 4.7.
14. Reporting bias assessment	Describe any methods used to assess risk of bias due to missing results in a synthesis.	Section 2.7 — not formally assessed; rationale given (fewer than ten studies per outcome, rendering funnel plot and regression methods uninformative).
15. Certainty assessment	Describe any methods used to assess certainty (or confidence) in the body of evidence for an outcome.	Not performed. Formal GRADE assessment was not undertaken; the limitations of the evidence base are described narratively in Sections 3.3 and 4.7.
RESULTS		
16a. Study selection	Describe the results of the search and selection process, from the number of records identified to the number of studies included, ideally using a flow diagram.	Section 3.1; Figure 1 (PRISMA flow diagram).
16b. Study selection (exclusions)	Cite studies that might appear to meet the inclusion criteria but which were excluded, and explain why they were excluded.	Section 3.1 (categories of exclusion at full-text stage, n = 18).
17. Study characteristics	Cite each included study and present its characteristics.	Section 3.2; Table 1.
18. Risk of bias in studies	Present assessments of risk of bias for each included study.	Section 3.3; Tables 2 and 3.
19. Results of individual studies	Present, for all outcomes, for each study: summary statistics for each group and an effect estimate with precision, ideally using structured tables or plots.	Figures 2–9 (forest plots displaying individual study estimates); Sections 3.4–3.8.
20a. Results of syntheses (study characteristics/risk of bias)	For each synthesis, briefly summarise the characteristics and risk of bias among contributing studies.	Sections 3.3–3.8.
20b. Results of syntheses (statistical synthesis)	Present results of all statistical syntheses conducted, including summary estimate and precision, and measures of heterogeneity.	Sections 3.4–3.8; Figures 2–9.
20c. Results of syntheses (heterogeneity)	Present results of all investigations of possible causes of heterogeneity among study results.	Sections 3.8, 4.3 and 4.7 (narrative).
20d. Results of syntheses (sensitivity analyses)	Present results of all sensitivity analyses conducted.	Not applicable — no sensitivity analyses were performed (see item 13f).
21. Reporting biases	Present assessments of risk of bias due to missing results for each synthesis assessed.	Not applicable — not formally assessed (see item 14); limitation stated in Section 4.7.
22. Certainty of evidence	Present assessments of certainty in the body of evidence for each outcome assessed.	Not applicable — formal certainty assessment not performed (see item 15).
DISCUSSION		
23a. Discussion (interpretation)	Provide a general interpretation of the results in the context of other evidence.	Sections 4.1–4.5.

23b. Discussion (limitations of evidence)	Discuss any limitations of the evidence included in the review.	Section 4.7.
23c. Discussion (limitations of review processes)	Discuss any limitations of the review processes used.	Section 4.7 (bivariate model not estimable; search date; no sensitivity or certainty assessment).
23d. Discussion (implications)	Discuss implications of the results for practice, policy and future research.	Sections 4.6 and 4.8.
OTHER INFORMATION		
24a. Registration and protocol	Provide registration information for the review, including register name and registration number, or state that the review was not registered.	Section 2.1 — PROSPERO CRD420251138324.
24b. Protocol access	Indicate where the review protocol can be accessed, or state that a protocol was not prepared.	Section 2.1 — accessible via the PROSPERO register entry.
24c. Amendments	Describe and explain any amendments to information provided at registration or in the protocol.	No amendments to the registered protocol were made.
25. Support	Describe sources of financial or non-financial support for the review, and the role of the funders or sponsors.	Funding section — no external funding received.
26. Competing interests	Declare any competing interests of review authors.	Conflicts of Interest section.
27. Availability of data, code and other materials	Report which of the following are publicly available and where they can be found: template data collection forms; data extracted from included studies; data used for all analyses; analytic code; any other materials used in the review.	Data Availability Statement. All analysed data are derived from the published articles cited and are presented within the article and supplementary material.
