# Supplementary material for: Diagnostic Accuracy and Renal Safety of Carbon Dioxide Angiography in Lower Limb Angiography: A Systematic Review and Meta-Analysis
Source: Diagnostics (Basel). 2026 Oct 14;16(20):3341. doi: 10.3390/diagnostics16203341 (PMC13702889; doi:10.3390/diagnostics16203341)
Supplement: Supplementary file 1 [file diagnostics-16-03341-s001.zip › diagnostics-4598927 Supplementary_S2_Search_Strategies.pdf]

Supplementary Material S2. Full Electronic Search Strategies

Databases searched: PubMed (MEDLINE), Scopus, Web of Science Core Collection. Date range: inception to July 2024. No language or publication-type limits were applied at the search stage. Reference lists of all included studies and of two previously published meta-analyses on related questions were hand-searched.

PubMed (MEDLINE)

#1 "carbon dioxide"[MeSH Terms] OR "carbon dioxide"[tiab] OR "CO2"[tiab] OR carboxyangiograph*[tiab]

#2 "angiography"[MeSH Terms] OR angiograph*[tiab] OR arteriograph*[tiab] OR "digital subtraction"[tiab] OR DSA[tiab]

#3 "peripheral arterial disease"[MeSH Terms] OR "peripheral arterial disease"[tiab] OR "peripheral artery disease"[tiab] OR "peripheral vascular disease"[tiab] OR PAD[tiab] OR PAOD[tiab] OR "lower limb"[tiab] OR "lower extremity"[tiab] OR "critical limb ischemia"[tiab] OR "chronic limb-threatening ischemia"[tiab]

#4 "contrast media"[MeSH Terms] OR "iodinated contrast"[tiab] OR "contrast medium"[tiab] OR "contrast media"[tiab] OR iohexol[tiab] OR iodixanol[tiab]

#5 #1 AND #2 AND #3

#6 #5 AND #4

Scopus

TITLE-ABS-KEY (("carbon dioxide" OR "CO2" OR carboxyangiograph*) AND (angiograph* OR arteriograph* OR "digital subtraction") AND ("peripheral arterial disease" OR "peripheral artery disease" OR "peripheral vascular disease" OR "lower limb" OR "lower extremity" OR "critical limb ischemia" OR "chronic limb-threatening ischemia"))

Web of Science Core Collection

TS = (("carbon dioxide" OR "CO2" OR carboxyangiograph*) AND (angiograph* OR arteriograph* OR "digital subtraction") AND ("peripheral arterial disease" OR "peripheral artery disease" OR "peripheral vascular disease" OR "lower limb" OR "lower extremity" OR "critical limb ischemia" OR "chronic limb-threatening ischemia"))

Records retrieved

**Database**	**Records retrieved**
PubMed	78
Scopus	84
Web of Science	36
Total before de-duplication	198
Duplicates removed	48
Records screened	150
